# Combination of Selenium and UVA Radiation Affects Growth and Phytochemicals of Broccoli Microgreens

**DOI:** 10.3390/molecules26154646

**Published:** 2021-07-31

**Authors:** Meifang Gao, Rui He, Rui Shi, Yamin Li, Shiwei Song, Yiting Zhang, Wei Su, Houcheng Liu

**Affiliations:** College of Horticulture, South China Agricultural University, Guangzhou 510642, China; gmf@stu.scau.edu.cn (M.G.); ruihe@stu.scau.edu.cn (R.H.); ruishi27@stu.scau.edu.cn (R.S.); yaminli@stu.scau.edu.cn (Y.L.); swsong@scau.edu.cn (S.S.); yitingzhang@scau.edu.cn (Y.Z.); susan_l@scau.edu.cn (W.S.)

**Keywords:** *Brassica oleracea* var. *italica*, microgreens, sodium selenite, UVA, bioactive compounds

## Abstract

Addition of selenium or application of ultraviolet A (UVA) radiation for crop production could be an effective way of producing phytochemical-rich food. This study was conducted to investigate the effects of selenium and UVA radiation, as well as their combination on growth and phytochemical contents in broccoli microgreens. There were three treatments: Se (100 μmol/L Na_2_SeO_3_), UVA (40 μmol/m^2^/s) and Se + UVA (with application of Se and UVA). The control (CK) was Se spraying-free and UVA radiation-free. Although treatment with Se or/and UVA inhibited plant growth of broccoli microgreens, results showed that phytochemical contents increased. Broccoli microgreens under the Se treatment had higher contents of total soluble sugars, total phenolic compounds, total flavonoids, ascorbic acid, Fe, and organic Se and had lower Zn content. The UVA treatment increased the contents of total chlorophylls, total soluble proteins, total phenolic compounds, and FRAP. However, the Se + UVA treatment displayed the most remarkable effect on the contents of total anthocyanins, glucoraphanin, total aliphatic glucosinolates, and total glucosinolates; here, significant interactions between Se and UVA were observed. This study provides valuable insights into the combinational selenium and UVA for improving the phytochemicals of microgreens grown in an artificial lighting plant factory.

## 1. Introduction

Broccoli (*Brassica oleracea* var. *italica*) has attracted particular attention due to its abundant bioactive compounds, such as phenolic compounds, ascorbic acid, mineral nutrition, and especially glucosinolates (GSLs) [1,2]. Microgreens, a kind of innovative vegetable that is usually cultivated under protected environments with artificial light or sunlight, require little or no fertilizer, as the seed provides adequate nutrition for its growth. Varying from vegetables’ variety, the period from seeding to harvest for microgreens might take one to three weeks [3]. Broccoli microgreens contain higher bioactive compounds than seeds or mature broccoli [4,5].

Selenium (Se) has been concerned for its effective biological functions, antioxidant and anticancer [6]. It is an essential trace element for animals and humans but not for plants [7]. However, plants can enrich Se and convert inorganic Se with certain toxicity such as selenite into relatively safe organic Se [8,9]. To meet the Se demand for human body, Se-fortified vegetables can be consumed. Additionally, it is an important means that phytoremediation with plants can absorb large amounts of selenium to solve the selenium pollution problem [10]. However, authors of previous literature postulated that selenocysteine was the major source of misincorporation into proteins [11]. Brassicaceae vegetables show higher nutritional value by Se fortification, for which not only Se content but also other phytochemicals increase. Se could significantly increase the contents of glucoraphanin (GRA), anthocyanins, flavonoids [12], phenolic acids [13], and mineral elements [14]. However, there were no significant effects of Se application on total GSLs accumulation in broccoli microgreens [8,9,12].

Ultraviolet A (UVA) (320–400 nm) could promote the accumulation of total GSLs in broccoli microgreens [15]. UVA accounts for 98.7% of the ultraviolet radiation reaching the Earth’s surface [16] and it is closed to the photosynthetic active spectrum (400 to 700 nm). UVA radiation stimulates phototropins and cryptochromes such as blue light [17], but it also causes different reactions in plants, which could be useful for improving plant quality without inhibiting growth such as kale [18,19], dropwort [20], lettuce [21], sowthistle [22], and mustard microgreens [23]. The application of UVA has been particularly effective in enhancing the phytochemicals in plants, but this effect was cultivar- or species-specific. UVA (380 nm, 6 μmol/m^2^/s) light had differential effects on red and green leaf lettuce metabolism; for example, supplemental UVA light increased nitrite contents in green leaf lettuce (6.4 times) but slightly reduced nitrite contents in red leaf lettuce [24]. Among basil, beet, and bok choy, a most positive and dominant effect of the supplemental UVA (366 nm, 12.4 μmol/m^2^/s) was observed on bok choy in 2,2–diphenyl–1–picrylhydrazyl free radical scavenging activity, the contents of total phenols, anthocyanins, ascorbic acid, and α-tocopherol [25].

However, there is currently no consensus about the effect of Se or UVA on the plant quality and there is limited information concerning the effect of the combination of Se and UVA on growth and phytochemical of vegetables. Furthermore, whether Se and UVA exist an interactive effect on the growth and phytochemicals contents of vegetables. In this study, therefore, we investigated the single and mutual effects of Se and UVA supplied on shoot growth, pigments, and bioactive compounds contents of broccoli microgreens. This study aims to providing valuable insights into the combinational Se and UVA for improving the phytochemicals of microgreens grown in an artificial lighting plant factory.

## 2. Results

### 2.1. Growth Characteristics

**Figure 1:**
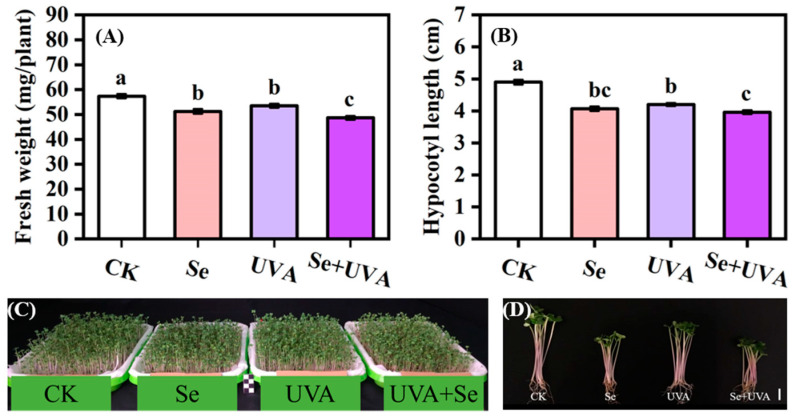
Fresh weight (**A**), hypocotyl length (**B**), and photos (**C, D**) of broccoli microgreens under Se (100 μmol/L Na_2_SeO_3_), UVA (40 μmol/m^2^/s), and combined treatments. The vertical bars indicated standard errors, *n* = 18 (**A**) or *n* = 39 (**B**). Different lower-case letters above the bars indicated significant differences between treatments by Duncan’s multiple range test at a level of 0.05. Photos of broccoli microgreens (**C, D**) were taken at 7 days after sowing, where the bars represent 1 cm.

Growth characteristics were significantly affected by the Se, UVA, and Se + UVA treatments. Fresh weight was negatively affected by Se (*p* < 0.001) and UVA (*p* < 0.01), whereas hypocotyl length was adversely influenced by Se (*p* < 0.001), UVA (*p* < 0.001), and the interaction of these two factors (*p* < 0.001) (Table 1). Compared with the control (CK), which was Se spraying-free and UVA radiation-free, the reduction in fresh weight of broccoli microgreens was found under the Se treatment (10.53%), the UVA treatment (5.26%), and the Se + UVA treatment (14.04%) (Figure 1A). Furthermore, the reductions in hypocotyl length of broccoli microgreens were found in the Se, the UVA, and the Se + UVA treatments (Figure 1B), which were 17.00%, 14.41%, and 19.28% shorter than CK, respectively.

### 2.2. Pigments Contents

**Figure 2:**
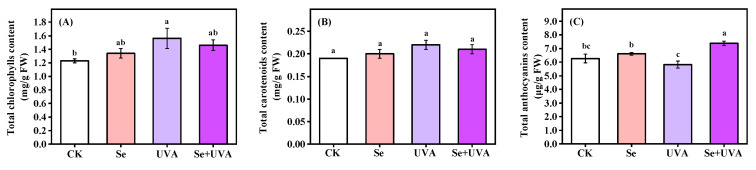
The contents of total chlorophylls (**A**), total carotenoids (**B**) and total anthocyanins (**C**) in broccoli microgreens under Se (100 μmol/L Na_2_SeO_3_), UVA (40 μmol/m^2^/s), and combined treatments. The vertical bars indicated standard errors, *n* = 3. Different lower-case letters above the bars indicated significant differences between treatments by Duncan’s multiple range test at a level of 0.05.

The total chlorophylls content of broccoli microgreens was affected by UVA radiation (*p* < 0.05) but not by exogenous Se (Figure 2A and Table 2) and was markedly higher (26.83%) in the UVA treatment than in CK (Figure 2A). However, total anthocyanins content was affected by Se (*p* < 0.05) but not by UVA, whereas there was a significant interaction between Se and UVA (*p* < 0.05) (Figure 2C and Table 2). The higher total anthocyanins content was only found in the Se + UVA treatment, 18% higher than that in CK (Figure 2C). There were no significant differences in total carotenoids content among all treatments (Figure 2B).

### 2.3. Primary Metabolite Content

**Figure 3:**
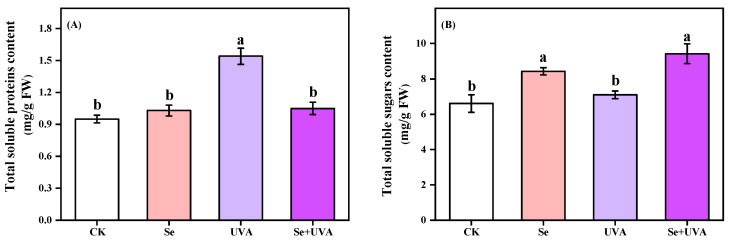
The contents of total soluble proteins (**A**) and total soluble sugars (**B**) in broccoli microgreens under Se (100 μmol/L Na_2_SeO_3_), UVA (40 μmol/m^2^/s), and combined treatments. The vertical bars indicated standard errors, *n* = 3. Different lower-case letters above the bars indicated significant differences between treatments by Duncan’s multiple range test at a level of 0.05.

Total soluble proteins content of broccoli microgreens was significantly affected by the UVA treatment (Figure 3A), which increased by 62.11%. However, the Se + UVA treatment did not show significant difference from CK in the content of total soluble proteins. Total soluble sugars content increased under the Se and the Se + UVA treatments. The Se treatment caused an increase of 20.86%, and the Se + UVA treatment increased by 26.45%, mainly due to Se application (*p* < 0.001) (Figure 3B and Table 3).

### 2.4. Antioxidant Content and Capacity

**Figure 4:**
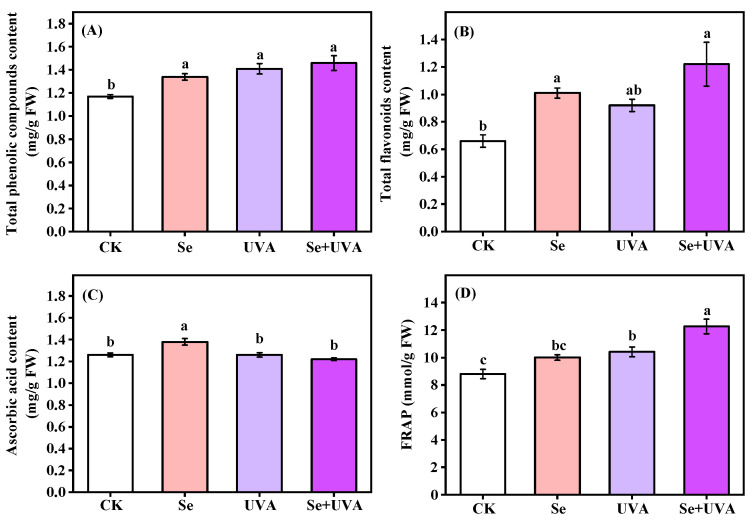
The contents of total phenolic compounds (**A**), total flavonoids (**B**), ascorbic acid (**C**), and FRAP (**D**) in broccoli microgreens under Se (100 μmol/L Na_2_SeO_3_), UVA (40 μmol/m^2^/s), and combined treatments. The vertical bars indicated standard errors, *n* = 3. Different lower-case letters above the bars indicated significant differences between treatments by Duncan’s multiple range test at a level of 0.05.

The antioxidant contents and capacity were affected by Se or/and UVA (Figure 4 and Table 4). The Se, UVA, and Se + UVA treatments led to significantly higher total phenolic compounds content in broccoli microgreens than CK, and the Se + UVA treatment presented the highest and increased by 24.79% (Figure 4A). The total flavonoids content increased under Se-treated microgreens; the Se treatment increased it by 53.03% and the Se + UVA treatment caused an increase of 84.85% (Figure 4B). The Se treatment presented higher ascorbic acid content than CK (Figure 4C). Simultaneous supplemental UVA radiation and Se treatment dramatically enhanced ferric reducing antioxidant power (FRAP) of broccoli microgreens following UVA treatment, increasing FRAP by 39.3% and 18.3%, respectively (Figure 4D).

### 2.5. Composition and Content of GSLs in Broccoli Microgreens

**Figure 5:**
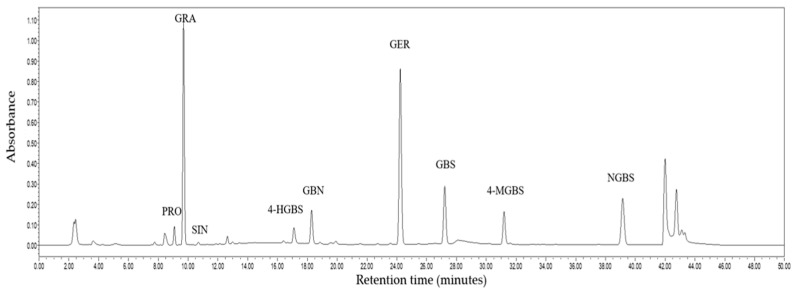
The composition of GSLs in broccoli microgreens. PRO, Progoitrin; GRA, glucoraphanin; SIN, sinigrin; 4-HGBS, 4-hydroxy-glucobrassicin; GBN, glucobrassicanapin; GER, glucoerucin; GBS, glucobrassicin; 4-MGBS, 4-methoxy-glucobrassicin; NGBS, neoglucobrassicin.

**Figure 6:**
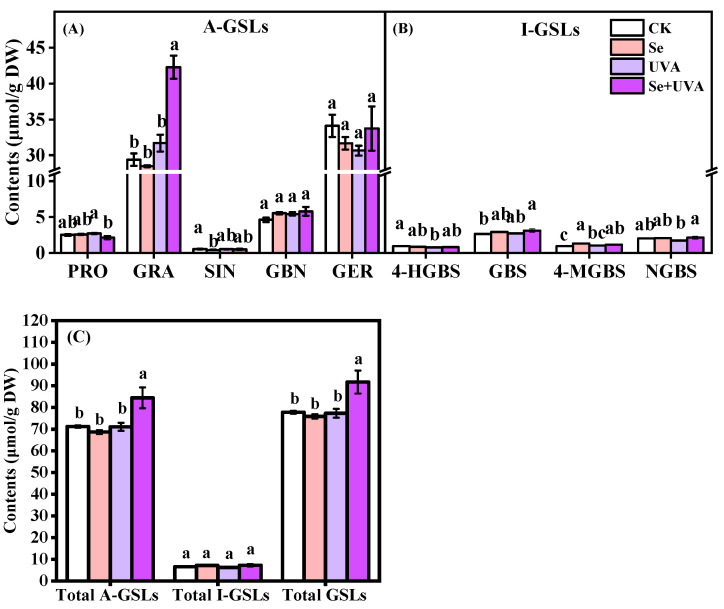
The contents of A-GSLs and I-GSLs content in broccoli microgreens under Se (100 μmol/L Na_2_SeO_3_), UVA (40 μmol/m^2^/s), and combined treatments (**A**–**C**). The vertical bars indicate standard errors, *n* = 3. Different lower-case letters above the bars indicate significant differences between treatments by Duncan’s multiple range test at a level of 0.05.

Night GSLs were identified in broccoli microgreens (Figure 5). The GSLs included five aliphatic glucosinolates (A-GSLs): progoitrin (PRO), glucoraphanin (GRA), sinigrin (SIN), glucobrassicanapin (GBN), and glucoerucin (GER), and four indolic glucosinolates (I-GSLs): 4-hydroxy-glucobrassicin (4-HGBS), glucobrassicin (GBS), 4-methoxy-glucobrassicin (4-MGBS), and neoglucobrassicin (NGBS). The A-GSLs were the predominant GSLs, accounting for more than 90% (Figure 6), and GRA and GER were the greater proportion of GSLs in all treatments (Figure 6).

Total A-GSLs showed the sum of the identified A-GSLs and total GSLs presented the sum of the identified GSLs. When compared with CK, the accumulation of total A-GSLs and total GSLs were obviously promoted in the Se + UVA treatment, which were significantly increased by 18.67% and 17.94% (Figure 6C), respectively. However, all treatments did not significantly affect the content of total I-GSLs (Figure 6C). Noticeably, the increment of total GSLs content in Se + UVA treatment were mainly due to the accumulation of GRA (Figure 6A,C). Higher GRA content (43.71%) and lower PRO content (16%) in broccoli microgreens were observed under the Se + UVA treatment in comparison with CK (Figure 6A). The Se treatment significantly decreased the SIN content, presenting a reduction of 23.78% compared with CK (Figure 6A). The contents of GBS and 4-MGBS in the Se + UVA treatment increased by 13.80% and 15.36%, respectively; the 4-MGBS content was 37.45% higher in the Se treatment than in CK (Figure 6B). All individual GSLs remained unaltered under the UVA treatment, but only 4-HGBS was significantly decreased by 19.79%, compared with CK (Figure 6A,B). Se significantly affected the contents of GRA (*p* < 0.01), GBS (*p* < 0.05), 4-MGBS (*p* < 0.01), and total I-GSLs (*p* < 0.05), whereas UVA significantly influenced the contents of GRA (*p* < 0.001), 4-HGBS (*p* < 0.05), total A-GSLs (*p* < 0.05), and total GSLs (*p* < 0.05) (Table 5). Significant interactions were observed between two factors (Se×UVA) for the contents of PRO (*p* < 0.05), GRA (*p* < 0.001), total A-GSLs (*p* < 0.05), and total GSLs (*p* < 0.05) (Table 5).

### 2.6. Mineral Elements of Broccoli Microgreens

**Figure 7:**
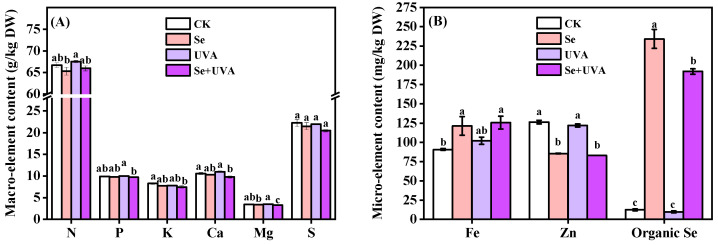
The contents of macroelement (**A**) and microelement (**B**) content in broccoli microgreens under Se (100 μmol/L Na_2_SeO_3_), UVA (40 μmol/m^2^/s), and combined treatments. The vertical bars indicate standard errors, *n* = 3. Different lower-case letters above the bars indicate significant differences between treatments by Duncan’s multiple range test at a level of 0.05.

No significant differences were detected in the mineral element contents under the UVA treatment; however, they were markedly differed under the Se and the Se + UVA treatments (Figure 7 and Table 6). There were no considerable differences in most of mineral elements between the Se and the Se + UVA treatments (Figure 7). The Se and Se + UVA treatments positively affected the contents of Fe (increased by up to 30%) and organic Se (increased up to 20-fold), while the Zn content in these treatments decreased by up to 30% compared with CK (Figure 7B). The lowest content of K, Ca, Mg, and S in broccoli microgreens were found under the Se + UVA treatment and decreased by nearly 10%, 8%, 6%, and 8%, respectively, compared with CK (Figure 7A), whereas significant interactions of Se and UVA (Se×UVA) were observed in the contents of Ca (*p* < 0.05) and Mg (*p* < 0.01) (Table 6). No striking differences in the contents of N, P, and S occurred in response to the three treatments (Se, UVA, and Se + UVA) in comparison with CK (Figure 7A).

### 2.7. Principal Component Analysis

**Figure 8:**
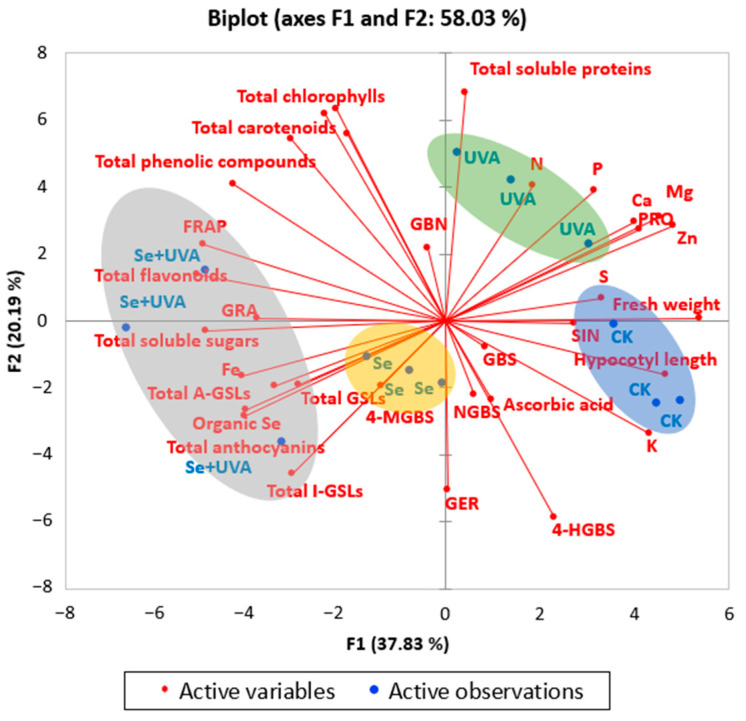
Loading plot and scores of PCA for fresh weight, hypocotyl length, total chlorophylls, total carotenoids, total anthocyanins, total soluble proteins, total soluble sugars, total phenolic compounds, total flavonoids, ascorbic acid, FRAP, total GSLs, total A-GSLs, total I-GSLs, individual GSL (PRO, GRA, SIN, 4-HGBS, GBN, GER, GBS, 4-MGBS, and NGBS), and minerals concentrations (N, P, K, Ca, Mg, S, Fe, Zn, and organic Se) in broccoli microgreens grown under Se and/or UVA treatment.

A principal component analysis (PCA) was conducted for all of the parameters measured above (Figure 8). The separation in PC1 was more pronounced and described about 34.37% of the variance, whereas PC2 only described about 20.17% of the data and separation of the groups was less clear. Variable plot shown in Figure 8 indicated that there were significant differences among CK, Se, UVA, and Se + UVA treatments. The Se treatment was mainly represented by contents of total soluble sugars and ascorbic acid; it was negatively correlated with growth parameters (fresh weight and hypocotyl length) and the contents of mineral element (N, P, S, Ca, Mg, and Zn). Meanwhile, the UVA treatment was mainly represented by the contents of total soluble proteins and mineral elements (N, P, S, Ca, Mg, and Zn). Additionally, the Se + UVA treatment was mainly represented by the contents of antioxidants (total phenolic compounds, total flavonoids, and FRAP), GSLs (GRA, GBS, and 4-MGBS) and two microelements (organic Se and Fe); it was negatively correlated with the two growth parameters. Variation in the two growth parameters was positively correlated with many minerals, including the contents of N, P, K, Ca, Mg, S, and Zn; it was negatively correlated with contents of total phenolic compounds, total soluble sugars, and total flavonoids. Furthermore, FRAP in broccoli microgreens showed positive correlations with the contents of total phenolic compounds and total flavonoids.

## 3. Discussion

### 3.1. Effects of Se and/or UVA Treatment on Growth of Broccoli Microgreens

Se could promote plant growth, but excessive selenium had a negative impact on the growth of plants [26,27]. A concentration of 5–30 mg/L Se in a solution did not affect the growth rate of the kale seedlings; however, 45 mg/L Se inhibited the growth rate [28]. According to PCA analysis, all treatments led to different reactions of plant (Figure 8). In broccoli microgreens, the fresh weight and hypocotyl length were significantly lower in the Se treatment than CK (Figure 1), suggesting that the concentration of 100 μmol/L (≈17 mg/L) Na_2_SeO_3_ might be too high for broccoli microgreens growth because it induces a decrease in the photosynthetic capacity [14], or because metabolic disruption due to the rapid conversion of inorganic Se into organic Se form led to inhibition of plant growth [29].

Compared with CK, the fresh weight and hypocotyl length of broccoli microgreens subjected to UVA (380 nm, 40 μmol/m^2^/s ≈ 10 W/m^2^) treatment also markedly decreased (Figure 1), similar to those of radish microgreens [30]. However, no inhibitory effects of UVA on the hypocotyls length were found in basil, beet, and bok choy microgreens [25]. Nevertheless, UVA (370 nm or 385 nm, 30 W/m^2^) LEDs induced significant increases in specific leaf weight, leaf area, and shoot fresh and dry weight of kale [18]. Supplemental UVA light (380 nm, 6 μmol/m^2^/s) superiorly stimulated plant growth of green leaf lettuce but resulted in diminished growth of the red leaf cultivar [24]. Plant growth response to UVA might be species- and/or cultivar-specific. The minimum fresh weight and hypocotyl length were observed in the Se + UVA treatment (Figure 1). Se and UVA had significant synergistic negative effects in detrimental to fresh weight without significant synergistic effects on the hypocotyl length (Table 1).

Mineral elements are vital for plant growth. Strong positive correlations between the elements (N, P, K, Ca, Mg, S, and Zn) and growth characteristics (fresh weight and hypocotyl length) were also observed in the loading plot and scores of PCA (Figure 8). Our experiments revealed that broccoli microgreens under the Se and Se + UVA treatments had the negative effect on growth and most of mineral element contents (except for the contents of Fe and organic Se) (Figure 7 and Figure 8).

### 3.2. Effects of Se or/and UVA Treatment on the Accumulation of Phytochemicals

Accumulating total chlorophylls content of broccoli microgreens under single UVA treatment (Figure 2A) might be due to the fact that UVA (380 nm) was closed to photosynthetically active radiation (PAR), which could be used as an additional and available source of light energy under low PAR [16]. However, Se combined with UVA did not affect the total chlorophylls content (Figure 2A) that Se might ease increase caused by UVA radiation. Similarly, UVA exposure caused increment in total chlorophylls content in broccoli sprouts, but UVA combined with methyl jasmonate caused reduction, which might be due to methyl jasmonate inducing stimulation of both ethylene formation and a symptom similar to aging where the photosynthetic electron transport rate and the *F_v_*/*F_m_* ratio decreased [31].

Se [32] and UVA [30,33] stimulated the accumulation of anthocyanins by inducing anthocyanin biosynthesis in the hypocotyls, which might occur due to the upregulated expression of phenylalanine ammonialyase (PAL). However, in this study, the Se or UVA treatment had no impact on the total anthocyanins content in broccoli microgreens (Figure 2C). The effect of Se and UVA on anthocyanins content in the broccoli sprouts might vary due to cultivars [12] and the levels of Se [32] and UVA [34], which need further studies. The contents of total soluble proteins and sugars in broccoli microgreens were remarkably improved by supplemental UVA and Se spraying alone, respectively (Figure 3A,B), as shown by PCA analysis (Figure 8), indicating that UVA was conducive to total soluble proteins accumulation and Se was conducive to total soluble sugars accumulation. Se treatment resulted in accumulation of total soluble sugars rather than total soluble proteins [35]. Pepper exposed to supplemental UVA radiation contained higher content of total proteins [36]. Supplying Se fertilizer improved total soluble sugars content in berries [37] and potato [38].

Mineral elements are also important for a wide variety of metabolic processes. Some nutrient elements play essential roles in the formation of chlorophylls. N and Mg are components of chlorophylls, whereas Fe and Zn have catalytic functions or other indirect effects in the biosynthesis of chlorophylls. Most of the cultivated plants possess the ability to absorb and store significant amount of Se in their tissues when grown on Se-enriched soils [39]. Se also increased most of mineral elements in rapeseed [14] and maize [40]. In our study, the Se and Se + UVA treatments enhanced contents of organic Se and Fe in broccoli microgreens and reduced the Zn content (Figure 7). These increases mainly contributed to the concentration of 17 mg/L Na_2_SeO_3_ (Table 6). In the shoot of maize, however, Fe accumulation was unaffected by inorganic Se treatment, whatever its form or concentration (from 0 to 1 mg/L) [41]. This might be due to that the Se treatment concentration was not high enough for maize to induce the change of Fe content. A negative effect on Zn was also observed in maize treated with selenite, because selenite inhibited the transport of Zn to shoots but not root uptake of maize [41]. Zinc deficiency in shoots might result in an increase in membrane permeability, thus inhibiting shoot growth [42]. No remarkable differences in the contents of Ca and Mg were found under the Se or UVA treatment, whereas significant reductions were observed under Se + UVA (Figure 7) and significant interactions between the two factors (Se×UVA) were observed on the contents of Ca (*p* < 0.05) and Mg (*p* < 0.01) (Table 6). These indicated that the interaction between selenium and UVA resulted in negative influences on calcium and magnesium metabolism.

In plants, due to the similar physicochemical properties, there is a common metabolic pathway between S and Se [10,40]. The Se and Se + UVA treatments had slight negative impact on the total S content in broccoli microgreens (Figure 7A), indicating that the metabolic pathway of selenium affected sulfur accumulation. Plants receiving a larger amount of UVA had more intensive mineral uptake in mustard microgreens [23]. Mineral elements in broccoli microgreens seemed to be unaffected by UVA (Figure 7), indicating that the 15 min irradiation of 40 μmol/m^2^/s UVA was not enough to stimulate mineral uptake in broccoli microgreens.

Plants protect themselves from abiotic and biotic stress by synthesizing phenolic compounds, flavonoids, ascorbic acid, and so on [43]. Phenolic compounds, flavonoids, and ascorbic acid play important roles in the antioxidant system [44,45,46]. Se and/or UVA induced the accumulation of total phenolic compounds in broccoli microgreens (Figure 4A). Similarly, Se [25,30] and UVA [47] were reported to induce the accumulation of phenolic compounds in microgreens, which should be attributed to the fact that exogenous Se and UVA significantly stimulated PAL activity [18,47]. Flavonoids, a representative antioxidant group closely involved in defense mechanisms against abiotic stress, varied in response to different stress factors. Higher flavonoid content were observed in the Se and Se + UVA treatments (Figure 4B), which might be due to the fact that Se induced oxidative stress and enhanced enzymes activity concerning flavonoids biosynthesis [39]. Se and UVA supplied simultaneously showed the highest content of total phenolic compounds and total flavonoids in broccoli microgreens (Figure 4). Exposure to supplementary UVA, the ascorbic acid content in spring barley cv. Barke (sensitive to oxidative stress) significantly decreased, but there was no difference in spring barley cv. Bojos (tolerant to oxidative stress) [43]. This might be since broccoli microgreens were tolerant to oxidative stress that UVA could not excite, whereas Se treatment could excite that oxidative stress. Significant enhancement of FRAP was observed under UVA treatments in broccoli microgreens. Homoplastically, in green butter lettuce, significant enhancement of FRAP was observed under end-of-day supplementary UVA [48]. Though red butter lettuce showed stronger FRAP than green butter lettuce, there was no significant enhancement of FRAP under end-of-day supplementary UVA in red butter lettuce [48].

### 3.3. Effects of Se and/or UVA Treatment on Composition and Contents of GSLs

Similar to previous studies [35], nine GSLs, including five A-GSLs and four I-GSLs in broccoli microgreens, were determined (Figure 6). GRA, one of the A-GSLs, is the dominant and most important GSL in broccoli [49,50,51] and can be processed into an anticancer compound sulforaphane, which is considered as one of the most promising anticancer substances [52], whereas PRO, one of the A-GSLs, causes liver and kidney abnormalities [53]. SIN can be degraded by myrosinase to allyl isothiocyanate, which is the source of the piquant taste of mustard [54]. The glucosinolates 4-HGBS, 4-MGBS, and NGBS are known as derivatives of GBS [55], which all belong to I-GSLs. GBS is converted into the intermediates 1-hydroxy-glucobrassicin (1-HGBS) or 4-HGBS, which can be converted to NGBS and 4-MGBS, respectively [56].

PRO, SIN, and GBS are contribute to the bitter taste of *Brassica* vegetables [57]. Without UVA radiation, Se significantly reduced the SIN content, while less changes were found in PRO and GBS; however, under UVA radiation, Se significantly reduced the PRO content, but SIN and GBS content remained unchanged (Figure 6A,B). Therefore, broccoli microgreens under Se treatments (Se and Se + UVA) would taste less bitter. Two Se treatments enhanced the content of 4-MGBS but not GBS and 4-HGBS (Figure 6B). The 4-HGBS content reduced by UVA radiation did not affect its precursor GBS content and its product 4-MGBS content (Figure 6B). These might be due to the gene transcripts such as *CYP81F2* involved in catalyzing the hydroxylation of GBS to 4-HGBS, which was more easily reduced by UVA [15].

There was no effect of Se or UVA on the total A-GSLs content, whereas higher total A-GSLs accumulation was found under the Se + UVA treatment in broccoli microgreens; the same was found for total GSLs content (Figure 6C). Interactive analysis indicated that Se and UVA had positive synergetic effects on GSLs accumulation in broccoli microgreens, especially GRA (*p* < 0.001) (Table 5). The Se + UVA treatment accounted for the highest content of total GSLs, GRA, GBS, and 4-MGBS and accounted for the lowest content of PRO (Figure 6A,B), suggesting that the combination of UVA radiation with Se treatment could promote the accumulation of beneficial glucosinolate and inhibit the synthesis of harmful glucosinolate. UVA radiation might induce signal transduction responses only when Se stress is presented; these signaling responses may couple with those induced by Se to favor the accumulation of 4-MGBS [31]. Combined Se with UVA could be more effective than single treatment in improving the nutritional value of broccoli microgreens. Previous study has shown that maximum accumulation of S occurs in Brassicaceae [58], which might be related to its rich in glucosinolates containing S and N. Another study thought that the Se treatment might affect the metabolism of S, thus affecting the content of S-containing compounds, such as isothiocyanate and dimethyl disulfide [59]. PCA analysis in our study revealed that in broccoli microgreens the contents of N and S were negatively correlated with the contents of total GSLs, total A-GSLs, total I-GSLs, GRA, and so on (Figure 8); namely, N and S uptake might be used to synthesize GSLs, reducing the accumulation of N and S (Figure 7A). The Se + UVA treatment seemed to play a positive role in that pathway.

## 4. Materials and Methods

### 4.1. Chemicals and Reagents

Coomassie brilliant blue G-250 and albumin bovine were purchased from Solarbio (Beijing, China). Anthrone and rutin trihydrate were purchased from Sinophaem (Beijing, China). Potassium nitrate and L-ascorbic acid were purchased from GCRF (Guangzhou, China). Gallic acid monohydrate was purchased from DaMao (Tianjin, China), 2,2-diphenyl-1-picrylhydrazyl was purchased from TCI (Shanghai, China), and 2,3,5-triphenyl tetrazolium chloride was purchased from Hefei Bomei (Hefei, China). Chemicals and reagents used for GSLs extraction and analysis were of liquid chromatography grade, unless otherwise stated. Sinigrin standard was purchased from Sigma-Aldrich (St. Louis, MO, USA). Sulfatase was purchased from Shanghai Yuanye Bio-Technology Co., Ltd. (Shanghai, China). The water used was obtained from the RO/BIO Water System (18.2 MW cm, EPED Corporation, Nanjing, China).

### 4.2. Plant Material Cultivation Conditions, and treatments

**Figure 9:**
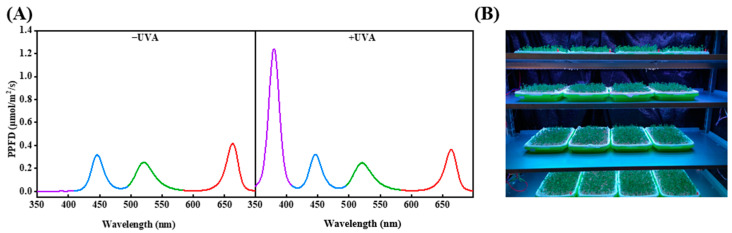
Spectra of light treatments (**A**) and photo of the experimental bench (**B**).

Broccoli seeds cv. lvhua (10 g per replication) were immersed in 5% sodium hypochlorite for 15 min and then drained. Next, seeds were rinsed in tap water and then soaked in tap water at 22 °C for 4 h. Afterward, the seeds were spread evenly on a tray (32.5 × 24 × 4.5 cm) filled with wet gauze and kept in darkness for 3 days, spraying tap water twice every day. On the fourth day after being sown, four treatments were conducted for 4 days: Se (40 mL 100 μmol/L Na_2_SeO_3_ solution spraying twice every day), UVA (380 ± 10 nm) (40 μmol /m^2^/s radiation for 15 min every day), Se + UVA, and CK (without Se and UVA application). Broccoli microgreens in CK and the UVA treatment were sprayed with 40 mL of purified water twice every day. Three repetitions were applied in this study. The environmental conditions in this experiment were as follows: 30 µmol/m^2^/s photosynthetic photon flux density (PPFD) with red:blue:green (1:1:1) light-emitting diodes, 16 h/8 h (light/dark), 22 ± 1 °C, relative humidity of 60% ± 10%, and 500 ± 100 µmol/mol CO_2_. The microgreens shoots were collected and sampled at the eighth day after they were sown. The samples were ground with mortar and pestle to fine powder in liquid nitrogen and stored in a freezer at −40 °C. Light spectra measured by a spectral analyzer (Lighting Passport ALP-01, AsenseTek, Taiwan) are shown in Figure 9A; the experimental bench is shown in Figure 9B.

### 4.3. Growth Characteristics

The shoot fresh weight (FW) and hypocotyl length of broccoli microgreens were determined by electronic balance and rectilinear scale. Eighteen replications were conducted with ten microgreens for fresh weight measurement and forty replications were conducted for hypocotyl length measurement.

### 4.4. Pigments Assays

The total chlorophylls content was measured according to the method used by Gratani [60]. Fresh leaf tissue (0.1 g) was soaked in 5 mL acetone/absolute ethanol mixture (*v:v* = 1:1) solution in dark until the color of leaf tissue became to colorless, and then total chlorophylls and total carotenoids were extracted. The extract liquor was measured at 645 nm, 663 nm, and 440 nm by a UV spectrophotometer (Shimadzu UV-16A, Shimadzu, Corporation, Kyoto, Japan). Pigments contents were calculated as follows:

Total chlorophylls content (mg/g FW) = (8.02 × A_663_ + 20.20 × A_645_) × 5 mL × 10^3^/ 0.1 g

Total carotenoids content (mg/g FW) = (4.7 × A_440_ − 0.27 × total chlorophylls content × 5 mL × 10^3^/ 0.1 g

The total anthocyanins content was measured as described by Xu et al. [61]. About 1.0 g microgreens were incubated for 2 h in 10 mL 60% methanol (pH = 3.0) at 60 °C water bath. Then, the solution was filtered and adjusted to 10 mL with 60% methanol. The absorbance at 535 nm was measured using the UV spectrophotometer (Shimadzu UV-16A, Shimadzu, Corporation, Kyoto, Japan). The results were presented as mg/g FW.

### 4.5. Primary Metabolites Assays

The total soluble proteins content was measured using Coomassie blue staining [62]. Fresh frozen tissue (0.5 g) was mixed with 4.0 mL distilled water and centrifuged at 3000 rpm for 10 min. The supernatant (0.2 mL) was diluted in the 0.8 mL distilled water and mixed well with 5.0 mL Coomassie brilliant blue G-250 solution. After 5 min, the mixture was measured at 595 nm using the UV spectrophotometer (Shimadzu UV-16A, Shimadzu, Corporation, Kyoto, Japan). Bovine serum albumin was used as a reference substance for the soluble proteins content analysis and the results were presented as mg/g FW.

The total soluble sugars content was measured using anthrone colorimetry [63]. Fresh frozen tissue (1.0 g) was mixed with 10 mL distilled water and boiled in a 100 °C water bath for 30 min. Again, approximately 10 mL of distilled water was added, boiled in a 100 °C water bath for 30 min, and then filtered. Then, 25 mL of distilled water was added. The solution (0.2 mL), distilled water (0.8 mL), anthrone ethyl acetate reagent (0.5 mL), and concentrated sulfuric acid (5 mL) were added in that order, mixing with vortex, and then placed in a boiling water bath for 10 min. After cooling to 25 °C, the solution was measured at 630 nm using the UV spectrophotometer (Shimadzu UV-16A, Shimadzu, Corporation, Kyoto, Japan). Glucose was used as a reference substance for the soluble sugars content analysis and the results were presented as mg/g FW.

### 4.6. Antioxidants Contents and Antioxidant Capacity Assays

The total phenolic compounds content was measured using the Folin–Ciocalteu colorimetric method [64]. Fresh frozen tissue (0.5 g) was extracted with 8.0 mL absolute ethanol. The sample extract (1.0 mL) was mixed well with 0.5 mL Folin–Ciocalteu reagent (diluted twice using deionized water), 1.5 mL 26.7% (*w*/*v*) sodium carbonate, and 7.0 mL distilled water. Finally, the mixture was left in darkness for 2 h. The absorbance at 760 nm was measured using the UV spectrophotometer (Shimadzu UV-16A, Shimadzu, Corporation, Kyoto, Japan). Gallic acid was used as a reference substance and the results were presented as mg/g FW.

The total flavonoids content was measured using an aluminum nitrate method [65]. Fresh frozen tissue (0.5 g) was extracted with absolute ethanol (8.0 mL). Broccoli microgreens extract (1.0 mL) was mixed with sodium nitrite solution (5%, 0.7 mL) for 5 min. Then, aluminum nitrate (10%, 0.7 mL) was added to the mixture for 6 min. Sodium hydroxide solution (5%, 5.0 mL) was added. The absorption at 510 nm was measured using the UV spectrophotometer (Shimadzu UV-16A, Shimadzu, Corporation, Kyoto, Japan). Rutin was used as a reference substance and the results were presented as mg/g FW.

The ascorbic acid content was measured using molybdenum blue spectrophotometry [66]. Fresh frozen tissue (0.5 g) was homogenized with 25 mL oxalic acid EDTA solution (*w*/*v*) and then filtered. 10 mL supernatant was mixed with 1 mL partial phosphoric acid-acetic acid solution (*w*/*v*) and 2 mL 5% sulphuric acid solution (*v*/*v*) and 4 mL 5% ammonium molybdate solution (*w*/*v*). The supernatants were mixed well and set still for 15 min; then, measured at 705 nm using the UV spectrophotometer (Shimadzu UV-16A, Shimadzu, Corporation, Kyoto, Japan). L-ascorbic acid was used as a reference substance for the ascorbic acid content analysis and the results were presented as mg/g FW.

FRAP was measured according to the procedure described by Benzie and Strain [67]. Fresh frozen tissue (0.5 g) was extracted by absolute ethanol (8.0 mL). The sample extract (0.4 mL) was added to 3.6 mL TPTZ solution (10 mmol/L TPTZ, 20 mmol/L ferric chloride and 300 mmol/L sodium acetate buffer (pH 3.6) at a ratio of 1:1:10 (*v:v:v*)). After 10 min incubation at 37 °C, absorption was measured at 593 nm using the UV spectrophotometer (Shimadzu UV-16A, Shimadzu, Corporation, Kyoto, Japan). FeSO_4_ was used as a reference substance and the results of antioxidant activities were presented as mmol/g FW.

### 4.7. GSLs Contents Assays

The fresh samples were dried for 48 h in a freeze-dry system (Songyuan Huaxing^®^, LGJ-18S, Beijing, China) and then stored at −40 °C. GSLs were extracted and analyzed as previously described [68]. Freeze-dried sample were extracted with methanol and then the extracts were purified and desulfurized with the ion-exchange method. The GSLs were separated and identified using high-performance liquid chromatography (Waters Alliance e2695, Waters Crop, Milliford, MA, USA). A 5 μm C18 column (250 mm length, 4.6 mm diameter, Waters, Milford, MA, USA) was used for GSLs separation. Elution was performed with mobile phase A (water) and mobile phase B (acetonitrile). The optimum column temperature was set at 30 °C. At a flow rate of 1 mL/minute, the gradient conditions were set as follows: solvent A volume at 100% for 0 to 32 min, 80% for 32 to 38 min, and solvent B volume at 100% for 38 to 40 min. The time for elution was 42–50 min. The detector monitored GSLs at 229 nm. Sinigrin was used as an internal standard for quantitation analysis and the results were presented as μmol/g DW (dry weight) of broccoli microgreens.

### 4.8. Determination of Elements Contents

Fresh samples were heated to deactivate enzyme at 105 °C for 2 h, then dried to a constant weight at 75 °C. They were smashed into power and stored. Dried powder (0.5 g) was analyzed for the content of N, P, K, Ca, Mg, S, Fe, Zn, and organic Se. Total N, P, and K was determined using Ojeda’s method [69], Mo-Sb colorimetry [70], and the flame photometry method [71], respectively; Ca, Mg, S, Zn, and Fe content were measured using the atomic absorption spectrophotometry method [72]. Organic Se content was measured as described by Li’s method [73].

### 4.9. Statistical Analysis

All the assays were analyzed in three replications (unless otherwise noted). The Shapiro–Wilk and Levene methods were used to verify the normal distribution and the homogeneity of variances, respectively, and when necessary, data were transformed subject to ANOVA. Normally distributed data meeting the assumption of homoscedasticity were analyzed using Duncan’s multiple-range tests at a level of 0.05. When normally distributed data did not meet the assumption of homoscedasticity, the Games–Howell post hoc test at a level of 0.05 was applied. One-way variance analysis with the Se or UVA factor and two-way analysis with the combination of Se and UVA was performed using SPSS 26.0 software (Chicago, IL, USA) to determine the significance at the levels of *p* < 0.05, 0.01, and 0.001. Principal component analysis (PCA) was performed using the XLSTAT 2019. The relationships between the paired variables were determined using Pearson correlation analysis at *p* < 0.05.

## 5. Conclusions

Though treatment with Se or/and UVA had a negative effect on plant growth of broccoli microgreens, they increased the phytochemicals contents. The Se, UVA, and Se + UVA treatments increased total phenolic compounds content and FRAP; however, only the UVA treatment accumulated the contents of chorophylls and total soluble proteins. Broccoli microgreens under the Se and Se + UVA exhibited higher contents of total soluble sugars, 4-MGBS, Fe, and organic Se and lower Zn content. In addition, there were significant interactions between supplemental Se and UVA; the Se + UVA treatment displayed the most remarkable effect on the contents of total anthocyanins, glucoraphanin, total aliphatic glucosinolates, and total glucosinolates. Therefore, combined sodium selenite spraying and UVA treatment would be a potential technology for improving the nutritional quality of microgreens. Future studies should focus on the selection of proper level of combined treatment for enhancing phytochemicals contents without inhibiting growth of microgreens.

## Figures and Tables

**Table 1:** The interaction effects of Se and UVA on growth characteristics of broccoli microgreens.

Interaction	Growth Characteristics	
	Fresh Weight	Hypocotyl Length
Se	***	***
UVA	**	***
Se × UVA	NS	***

NS, ** and *** represent nonsignificant or significant at *p* < 0.05, 0.01, and 0.001, respectively, according to two-way analysis of variance (ANOVA).

**Table 2:** The interaction effects of Se and UVA on pigments contents of broccoli microgreens.

Interaction	Pigments Contents		
	Total Chlorophylls	Total Carotenoids	Total Anthocyanins
Se	NS	NS	*
UVA	*	NS	NS
Se × UVA	NS	NS	*

NS and * represent nonsignificant or significant at *p* < 0.05, according to two-way ANOVA.

**Table 3:** The interaction effects of Se and UVA on primary metabolite content of broccoli microgreens.

Interaction	Primary Metabolite Content	
	Total Soluble Proteins	Total Soluble Sugars
Se	**	***
UVA	***	NS
Se × UVA	***	NS

NS, **, and *** represent nonsignificant or significant at *p* < 0.01 and 0.001, respectively, according to two-way ANOVA.

**Table 4:** The interaction effects of Se and UVA on antioxidant content and capacity of broccoli microgreens.

Interaction	Antioxidant Content and Capacity			
	Total phenolic Compounds	Total Flavonoids	Ascorbic Acid	FRAP
Se	*	**	NS	**
UVA	**	*	**	***
Se × UVA	NS	NS	**	NS

NS, *, **, and *** represent nonsignificant or significant at *p* < 0.05, 0.01, and 0.001, respectively, according to two-way ANOVA.

**Table 5:** The interaction effects of Se and UVA on GSLs contents of broccoli microgreens.

Interaction	PRO	GRA	SIN	GBN	GER	4-HGBS	GBS	4-MGBS	NGBS	Total A-GSLs	Total I-GSLs	Total GSLs
Se	NS	**	NS	NS	NS	NS	*	**	NS	NS	*	NS
UVA	NS	***	NS	NS	NS	*	NS	NS	NS	*	NS	*
Se×UVA	*	***	NS	NS	NS	NS	NS	NS	NS	*	NS	*

NS, *, **, and *** represent nonsignificant or significant at *p* < 0.05, 0.01, and 0.001, respectively, according to two-way ANOVA.

**Table 6:** The interaction effects of Se and UVA on mineral elements content of broccoli microgreens.

Interaction	Macroelement						Microelement		
	N	P	K	Ca	Mg	S	Fe	Zn	Organic Se
Se	*	*	NS	**	***	NS	***	**	***
UVA	NS	NS	NS	NS	NS	NS	NS	NS	NS
Se × UVA	NS	NS	NS	*	**	NS	NS	NS	NS

NS, *, **, and *** represent nonsignificant or significant at *p* < 0.05, 0.01, and 0.001, respectively, according to two-way ANOVA.

## Sample Availability

Samples of the compounds are available from the authors.
